# Postmortem study of adrenomedullin and cortisol in femoral serum and pericardial fluid related to acute pulmonary edema

**DOI:** 10.1007/s00414-024-03337-6

**Published:** 2024-09-26

**Authors:** Daniel Martínez-Jiménez, Juan Pedro Hernández del Rincón, Maria Sabater-Molina, Cristina Pérez-Martínez, Carmen Torres, María D. Pérez-Cárceles, Aurelio Luna

**Affiliations:** 1 Department of Legal and Forensic Medicine, University of Murcia, Campus Ciencias de la Salud Cmno Buenavista s/n 30120 El Palmar, Murcia, Spain; 2 Institute of Legal Medicine and Forensic Sciences of Murcia, Murcia, Spain; 3 Biomedical Research Institute of Murcia (IMIB), Murcia, Spain

**Keywords:** Physiopathological mechanisms, Biomarkers, Acute pulmonary edema, MR-proADM, Cortisol

## Abstract

Currently, various tools aid in determining the cause of death and the circumstances surrounding it. Thanatochemistry is one such method that provides insights into the physiopathological mechanisms of death and the behavior of specific biomarkers in different body fluids postmortem. Certain biomarkers, characterized by their stability and specificity to vital tissues like the lungs, are associated with mechanisms contributing to death, such as acute pulmonary edema (APE). This study aims to analyze the behavior of midregional pro-adrenomedullin (MR-proADM) and cortisol levels, measured in pericardial fluid and femoral serum, in relation to the severity of APE, categorized according to specific criteria. Samples were collected from a total of 92 corpses (77 males, 15 females) with a mean age of 56.7 ± 15.2 years. The severity of APE associated with the deaths was classified into three groups: slight or absent (*n* = 7; 8.6%), medium or moderate (*n* = 16; 19.8%), and intense (*n* = 58;71.6%).

The determination of MR-proADM and cortisol levels was conducted using ELISA kits and an Immunoassay Analyzer, respectively. Our results reveal a significant increase in MR-proADM concentration with the severity of APE. Furthermore, a correlation was established between cortisol and MR-proADM concentrations in both pericardial fluid and femoral serum samples. This indicates that the severity of APE influences the production of ADM, regardless of the specific underlying pathophysiological mechanisms. Cortisol values were also found to be higher in the intense APE group compared to the moderate group.

This study contributes to our understanding of the relationship between MR-proADM and cortisol, and the severity of APE, shedding light on potential applications in postmortem investigations.

## Introduction

In legal medicine, thanatochemistry has evolved into a method of supporting the diagnosis of the cause of death and the circumstances surrounding it. It also enables the pathological state of the person *antemortem* to be evaluated and provides valuable information on the physiopathological mechanisms of death [1–3]. This diagnosis method studies the behavior and stability of several biochemical postmortem markers of body fluids such as cerebrospinal fluid (CSF), pericardial fluid, serum-plasma or vitreous humour. Given that these fluids are compartmentalized, assessing alterations or contamination after death poses significant challenges [3].

Additional challenges include the lack of comprehensive databases for biochemical elements, which complicates interpretation and differentiation between pathological and normal states [2], as well as variations in parameters due to the agonic process and postmortem changes [3–6]. Factors influencing cellular alterations can be extrinsic, such as ambient temperature and isolation of the corpse, or intrinsic, like body fat and overall size of the deceased [7]. These factors can affect the kinetics of decomposition [8], leading to changes in biochemical parameters after death [9].

Among the most studied biochemical parameters due to their relative stability are urea nitrogen, serum proteins, cholesterol, bilirubin, cholinesterase, C-reactive protein (CRP), erythropoietin, and hemoglobin A1C. Other parameters, such as creatinine, uric acid, amylase, gamma-glutamyl transferase (GGT), pulmonary surfactants, catecholamines, S100, and neopterin, increase during the agonic period or postmortem. Some markers, including magnesium and myocardial markers, can vary according to the cause of death, highlighting their role as indicators of vital tissues affected by fatal damage – such as the heart, lungs, and brain – with examples including troponin I and brain natriuretic peptide (BNP) [5–11].

Acute pulmonary edema (APE) is a common mechanism of death, and several hypotheses have been proposed to explain it, including alveolar damage and cardiogenic, hemodynamic, and neurogenic mechanisms [12, 13]. These are classified as: (i) non-cardiogenic APE, related to conditions such as drowning, fluid overload, aspiration, inhalation injuries, neurogenic pulmonary edema, acute renal illness, allergic reactions, and adult respiratory distress syndrome; and (ii) cardiogenic APE, associated with high pressures in the left atrium due to factors like mitral stenosis, left ventricular dysfunction, and myocardial infarction [14].

Adrenomedullin, a biomarker of cardiac injury, is crucial in assessing the severity of heart failure and regulating cardiovascular homeostasis. Its role in cardiac tissue remodeling, particularly in myocardial hypertrophy, has been extensively studied [15]. Midregional pro-adrenomedullin (MR-proADM), an indicator of hypoxia, is relevant in the context of APE and is involved in increased vascular permeability in both heart failure and severe hypoxia [16–18]. Cortisol, another critical biomarker, is valuable in forensic practice for assessing adrenal response during agonal suffering. Elevated cortisol levels in cases of sudden death related to stress reflect this adaptive mechanism and can help indicate acute stress during the autopsy [19]. Furthermore, cortisol levels, ranging from 8 to 27 micrograms/dl, remain stable during the post-mortem period, as cortisol is not significantly affected by autolysis [20].

The aim of our study was to determine the levels of MR-proADM and cortisol in postmortem cases and their relationship with the severity of acute pulmonary edema according to these parameters.

## Materials and methods

### Sample collection

This was a prospective cohort study of consecutive autopsies conducted from January 2019 to December 2021. The samples were collected 2 to 48 h post-mortem, with an average of 17.5 ± 8.4 h, during routine forensic autopsies. The rationale for conducting biochemical studies within this timeframe is to minimize potential alterations caused by analytical interferences that may occur during the postmortem period and to obtain results that can serve as a complementary tool in determining the cause of death. Pericardial fluid was carefully extracted via a small incision in the pericardium to avoid contamination. Femoral blood was drawn by puncturing the femoral vein in the inguinal area. The collected samples were centrifuged at 3000 rpm for 10 min, and the resultant plasma and serum were stored at -80 °C until analysis. The study received approval from the Local Ethics Committees and adhered to the principles of the Declaration of Helsinki. The authors ensure the integrity of the data.

### Inclusion/Exclusion criteria and post-mortem examinations

All autopsies were performed by experienced forensic pathologists with specific training in both anatomical and histopathological examinations. The criteria for including cases were based on availability of complete medical records and autopsy findings, while cases with incomplete data or significant post-mortem damage were excluded. The post-mortem interval (PMI) was estimated based on the time elapsed from the confirmed time of death to the time of autopsy.

### Analysis criteria

To evaluate the biochemical parameters, autopsy samples were classified into five groups based on the etiology of death. This classification was determined from patient medical records, the scene of death observations, autopsy findings, and complementary toxicological and histological studies, as detailed in Table 1.

**Table 1:** Classification of individuals based on the etiology of death

Etiology of the death	Male	Female	Total	Contents
Myocardial infarction	27 (35.1)	4 (26.78)	31 (33.7)	Acute and chronic ischaemic heart disease Acute myocardial infarction Dilated cardiomyopathy
Traumatism	16 (20.8)	5 (33.3)	21 (22.8)	Brain injuries (Pedestrian impact, Bus, Fall, Traffic, Cervical injuries) Thoracic injuries Multiple trauma- hemoperitoneum
Violent asphyxias	16 (20.8)	1 (6.7)	17 (18.5)	Hanging Drowning
Intoxications	7 (9.1)	0 (0.0)	7 (7.6)	Hanging Drowning
Miscellaneous	11 (14.3)	5 (33.3)	16 (17.4)	Steiner disease (muscle disease) Lung abscess Chronic obstructive pulmonary disease Brain haemorrhage Gastrointestinal bleeding Respiratory failure Arrhythmogenic right ventricular cardiomyopathy Brain ruptured aneurysm Gunshot wound
Total	77 (83.7)	15 (16.3)	92 (100.0)	

Values are n (%)

APEs were classified according to the analysis of image and weight of lungs as light or absent, medium ormoderate and intense(Table 2).

**Table 2:** Criteria for classification of acute pulmonary edema

Acute pulmonary edema	Image of lungs	Weight
Slight or absent	Macroscopic and microscopic images within limits of normality	Weight of lungs normal, according to height and total weight of subject.
Medium or moderate	Image of any fluid present bronchioles and alveoli, that does not block the ducts to bronchioles	Any increase in weight does not exceed 25% of normal weight of normal lung.
Intense	Abundant secretion, presence of fluid that occludes the ducts from bronchioles and alveoli.	Increase in normal weight of normal lung exceeds 25%.

### Biochemical analysis

MR-proADM levels in plasma and serum were quantified using a Human MR-proADM ELISA kit (My Biosource, San Diego, CA), following the manufacturer’s instructions. Absorbance was measured at 450 nm on a DYNEX DS2^®^ (Dynex Magellan Biosciences, Chantilly, VA, USA). Cortisol levels were assessed using Abbott CMIA kits on an Abbott Architect i6000 Immunoassay Analyzer, ensuring a run precision of approximately CV 4% and a total CV of 5%.

### Statistical analysis

Data are presented as medians with interquartile ranges (IQR) or as counts and percentages where appropriate. The normality of distributions was verified using the Kolmogorov-Smirnov test. Continuous variables were analyzed using the Student’s t-test or Mann-Whitney U test, and the Kruskal-Wallis test was employed for comparisons across multiple groups. Spearman’s coefficient was used for correlational analyses. A significance level of *p* ≤ 0.05 was adopted for all tests. Statistical analyses were performed using SPSS v.20.0 (IBM SPSS Statistics, Inc., Chicago, IL, USA).

## Results

Samples of pericardial fluid and peripheral blood were collected from 92 individuals, comprising 77 males (83.7%) and 15 females (16.3%), with ages ranging from 17 to 88 years (mean age 56.7 ± 15.2 years). The subjects were categorized by the cause of death into five groups: myocardial infarction (*n* = 31), traumatism (*n* = 21), violent asphyxias (*n* = 17), intoxications (*n* = 7), and miscellaneous (*n* = 16). No statistically significant differences were found in the demographic or biochemical parameters across these categories (Table 3), suggesting that the primary categorization was not a determinant in the variations observed in our measured markers. Nevertheless the males were significantly predominant in the five groups.

**Table 3:** Demographic data and concentrations of biochemical parameters as a function of etiology of death

		Cause of death					*P* value
		Myocardial infarction (*n* = 31)	Traumatism (*n* = 21)	Violent asphyxias (*n* = 17)	Intoxications (*n* = 7)	Miscellaneous (*n* = 16)	
Gender	Male (*n* = 77)	27 (35.1)	16 (20.8)	16 (20.8)	7 (9.1)	11 (14.3)	0.167
PMI (hrs)		15.8 ± 9.3	16.5 ± 7.0	19.6 ± 8.5	20.0 ± 7.0	18.6 ± 9.0	0.490
Age (years)		61.2 ± 11.6	58.0 ± 18.9	51.4 ± 17.2	49.0 ± 14.7	55.1 ± 12.4	0.138
APE		29 (35.8)	18 (22.2)	15 (18.5)	7 (8.6)	12 (14.8)	0.412
	Slight or absent	1 (3.4)	3 (16.7)	1 (6.7)	0 (0.0)	2 (16.7)	0.383
	Medium or moderate	6 (20.7)	5 (27.8)	2 (13.3)	0 (0.0)	3 (25.0)	0.546
	Intense	22 (75.9)	10 (55.6)	12 (80.0)	7 (100.0)	7 (58.3)	0.143
ADM (ng/L)	FS	1842.7 (630.5-3980.1)	1694.5 (719.0-4004.0)	2040.7 (966.2-3446.5)	2294.0 (1226.5–4104.0)	1512.0 (323.5-2771.5)	0.710
	PF	2116.5 (614.0-2871.5)	1899.0 (289.0-3714.8)	1768.9 (1071.3-3593.5)	2141.5(1641.5-2906.5)	1768.9 (1406.0-4220.2)	0.531
Cortisol(ng/L)	FS	12.1 (1.7–62)	8.6 (1.6–76.0)	8.0 (4.6–15.9)	17.0 (7.3–20.4)	10.8 (3.2–24.0)	0.250
	PF	10.7 (2.1–75.0)	8.9 (2.1–31.0)	10.4 (5.7–13.1)	14.1 (4.2–18.3)	10.9 (1.2–19.9)	0.836

Values are n (%),mean ± S.D or median (interquartile range)

APE: Acute pulmonary edema; PMI: Postmortem interval; ADM: Adrenomedullin; IQR: Interquartile range; FS: Femoral serum; PF: Pericardial fluid

The individuals were also classified according to APE severity into three groups: slight or absent (*n* = 7; 8.6%), medium or moderate(*n* = 16; 19.8%) and intense (*n* = 58;71.6%) (Table 4). Of the 81 cases of APE, 43.2% (*n* = 35) were cardiogenic and 56.8% (*n* = 46) non-cardiogenic.

**Table 4:** Levels of in the diagnostic groups

Biomarkers (*N* = 81)	APE: absent (*n* = 7)	APE: moderate (*n* = 16)	APE: intense (*n* = 58)	*p* value
Gender(Male, 67)	5 (71.4)	11 (68.8)	51 (87.9)	0.142
Age (yr)	60.0 ± 14.2	56.6 ± 15.7	55.8 ± 15.4	0.794
PMI (hrs)	24,7 ± 10,7*	17,0 ± 10,1	16,5 ± 7,4*	0.05 (0.045)*
Cardiogenic	4 (57.1)	7 (43.8)	24 (41.4)	0.728
ADM FS (ng/L)	521.2 (323.5–719.0)	1195.2 (630.5-1647.5)	2143.6(966.2–4104.0)	< 0.0001
ADM PF (ng/L)	2254.6 (289.0-4220.2)	1336.3 (614.0-3051.5)*	2169.0 (926.0-3593.5)*	0.109 (0.028)*
Cortisol FS (μg/L)	13.9 (9.5–18.4)	7.0 (3.8–12.0)*	8.8 (1.6–24.0)*	0.071 (0.047)*
Cortisol PF (μg/L)	13.6 (11.5–15.8)	9.0(5.8–11.1)	9.7 (1.2–19.9)	0.189

Values are n (%),mean ± S.D or median (interquartile range).APE: Acute pulmonary edema; PMI: Postmortem interval; ADM: Adrenomedullin; FS: Femoral serum; PF: Pericardial fluid

*Significant differences between groups

**Fig. 1:**
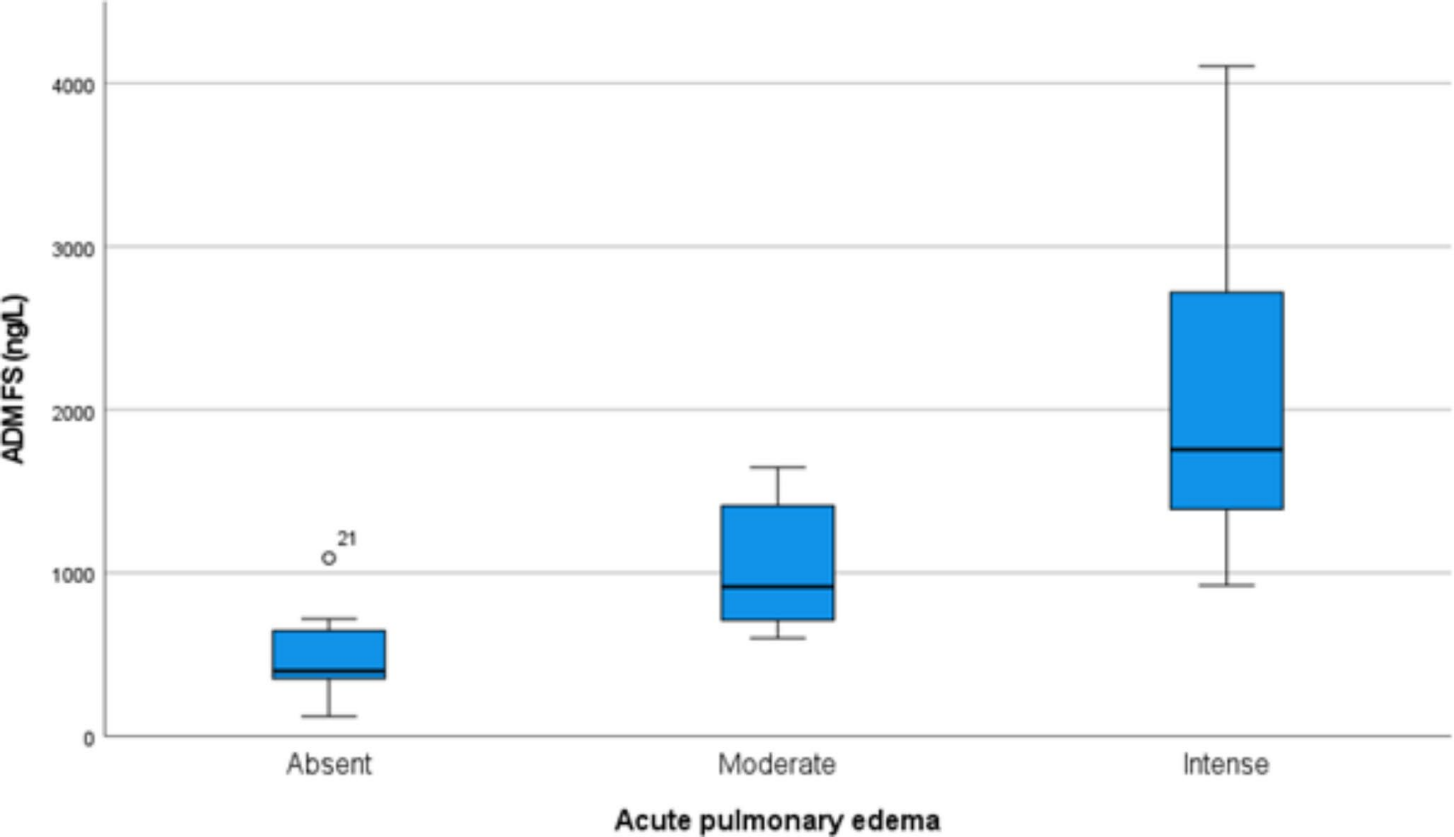
Femoral serum ADM levels across different groups based on Acute Pulmonary Edema (APE) severity

In the study of postmortem biomarkers for APE, we explored the correlations of ADM and cortisol levels in femoral serum and pericardial fluid with the severity of APE. Our findings demonstrated a significant increase in ADM levels in femoral serum correlated with escalating severity of APE, showing a marked statistical significance (*p* < 0.0001) (Fig. 1). Additionally, when comparing groups with intense APE to those with moderate APE, ADM levels in pericardial fluid also increased significantly (*p* = 0.028). Cortisol concentrations in femoral serum were higher in the intense APE group compared to the moderate group (*p* = 0.047), further supporting the utility of these biomarkers in gauging the severity of APE.

In terms of biomarker interrelationships, adrenomedullin and cortisol exhibited a significant correlation in both femoral serum (*r* = 0.244, *p* = 0.031) and pericardial fluid (*r* = 0.732, *p* < 0.001). This suggests a consistent pattern across different biological matrices, indicating that these biomarkers may reflect underlying pathophysiological processes associated with APE severity cohesively.

Moreover, our analysis revealed significant correlations between cortisol levels in both femoral serum (*r* = 0.312, *p* = 0.005) and pericardial fluid (*r* = 0.409, *p* = 0.001) with the post-mortem interval (PMI). Interestingly, cortisol concentrations were notably higher in cases absent of APE. This finding may implicate cortisol’s role not only as a marker of stress response but also its potential variability influenced by the PMI and the presence or absence of acute pulmonary pathologies.

These results underscore the potential of ADM and cortisol as valuable biomarkers for determining the severity of APE in postmortem examinations. They also highlight the complexity of interpreting such markers, where factors like PMI must be carefully considered to optimize the accuracy of forensic investigations. Further studies are recommended to validate these findings and expand our understanding of the biochemical changes that occur after death, particularly in cases of pulmonary distress.

## Discussion

In forensic investigations, especially those involving sudden, unexpected, or traumatic deaths, a comprehensive medico-legal evaluation is crucial to uncovering the underlying causes and mechanisms of death [21]. Biochemical markers are vital in distinguishing different etiologies, offering insights that are pivotal in the medico-legal context.

This study focused on cortisol and MR-proADM, analyzing their levels in pericardial fluid and peripheral blood. Cortisol, a primary glucocorticoid, mediates the body’s response to stress [21], while MR-proADM plays a protective role in cellular stress responses and infection defense mechanisms [22, 23]. We investigated these biomarkers in relation to the severity of pulmonary edema (APE), categorizing it as absent, moderate, or intense.

Our findings indicate that ADM levels in femoral serum significantly increase with the severity of APE (*p* < 0.001), with the highest concentrations observed in cases with intense APE. This aligns with existing literature that shows elevated ADM levels in conditions characterized by significant fluid accumulation and severe peripheral edema [24–29]. ADM is particularly noted for its diagnostic clarity in congestive scenarios, often surpassing natriuretic peptides in heart failure [15, 30]. Its release in response to hypoxia and stress-related conditions such as sepsis and myocardial infarction, alongside its significant receptor expression in the lungs, further supports its role as a valuable biomarker [31–33].

Research on post-mortem cortisol levels remains sparse; however, our results suggest that cortisol concentrations vary with the intensity of APE across different aetiologies, with the highest levels observed in cases with intense APE. Interestingly, elevated cortisol levels in the absence of APE correlate strongly with longer post-mortem intervals (PMI), implying significant physiological stress or an extended PMI prior to death.

These data underscore the influence of APE on ADM and cortisol production, suggesting that the specific cause of death or the nature of APE (cardiogenic vs. non-cardiogenic) may not significantly affect these biomarker concentrations.

Elevated stress marker levels have also been observed in other fatal conditions, such as severe trauma, burns, major surgeries, hypoglycemia, fever, fluctuations in blood pressure, physical exertion, and exposure to extreme cold, further underscoring the complexity of post-mortem biochemical evaluations [34–39].

Importantly, our study highlights how MR-proADM and cortisol serve as dynamic biomarkers that offer insights into physiological suffering not always evident from histopathological examination alone. These biomarkers reflect the biochemical dynamics of APE, providing a broader understanding of the physiological stress experienced prior to death. They complement histopathological findings by adding a layer of dynamic biochemical information that can enhance the forensic assessment of death.

Overall, these findings underscore the potential of ADM and cortisol as valuable tools in forensic investigations, offering additional perspectives on the physiological processes at play and improving the interpretation of cause and manner of death in complex cases.

## Conclusions

This study demonstrates that serum levels of adrenomedullin (ADM) and cortisol correlate with the severity of acute pulmonary edema (APE) and the post-mortem interval (PMI). These biomarkers show potential as complementary tools to histopathological examination in postmortem studies. By providing additional insights into physiological conditions before death, ADM and cortisol can enhance forensic assessments and aid in the determination of the underlying causes of death.

## Data Availability

The data underlying this article are available in the article.
